# A home-based approach to managing multi-drug resistant tuberculosis in Uganda: a case report

**DOI:** 10.1186/1742-6405-9-12

**Published:** 2012-04-23

**Authors:** Emmanuel Luyirika, Henry Nsobya, Richard Batamwita, Pheona Busingye, William Musoke, Lillian Nabiddo, Yvonne Karamagi, Barbara Mukasa

**Affiliations:** 1 Mildmay Centre, PO Box 24985, Kampala, Uganda

**Keywords:** HIV, Tuberculosis, Multi-drug resistant, MDR-TB, Home-care, Home-based care, East Africa, Sub-Saharan Africa

## Abstract

This case report describes an HIV-positive patient with recurrent tuberculosis in Uganda. After several failed courses of treatment, the patient was diagnosed with multi-drug resistant tuberculosis (MDR-TB). As adequate in-patient facilities were unavailable, we advised the patient to remain at home, and he received treatment at home via his family and a community nurse. The patient had a successful clearance of tuberculosis. This strategy of home-based care represents an important opportunity for treatment of patients in East Africa, where human resource constraints and inadequate hospital facilities exist for complex patients at high risk of infection to others.

## Background

Successful treatment of multi-drug resistant tuberculosis (MDR-TB) in HIV-positive patients is a challenge in resource-limited settings. In Uganda, the exact magnitude of MDR-TB is not known, though a national drug resistance survey is ongoing. To date, there has not been any reported successful treatment of MDR-TB using the home-based approach in East Africa. However, a recent study conducted in South Africa found that a similar approach has been effective [1]. In a first attempt, Mildmay Uganda has successfully treated a patient with MDR-TB using a home-based approach.

## Case presentation

A 42-year old HIV-positive male, in a discordant relationship, presented to the Mildmay Uganda clinic with a long-standing cough, chest and abdominal pain, night sweats, and impairment of short-term memory. The patient had two previous episodes of pulmonary tuberculosis (TB), and on both occasions, this condition was managed in hospital, and he completed treatment in accordance with the Uganda Ministry of Health TB program standard regimes (2RHZE/6EH and 2RHZES/1RHZE/5RHE) [2]. On examination in March 2007, the patient had consolidation of the upper lobe of the left lung. Investigations showed the sputum ZN smear positive for AFBs (3+). A diagnosis of TB was made, and the patient started TB treatment (2RHEZS/1REHZ/5REH), as per standard guidelines [2]. A sputum sample was sent to the Ugandan National TB laboratory for mycobacterium culture; results indicated innumerable mycobacterium colonies, but unfortunately the laboratory could not perform drug sensitivity tests at that time. Follow-up ZN sputum smears performed 3 months after the patient started treatment were still positive for AFBs.

After 8 months on treatment, the patient's sputum was still positive for AFB. In April 2008, sputum cultures were sent to the mycobacteriology laboratory at the Joint Clinical Research Centre in Kampala, and results indicated mycobacterium TB resistant to the following TB drugs: streptomycin, isoniazid, and rifampicin. Colonies showed drug sensitivity to capreomycin, kanamycin, p-amino salicyclic acid (PAS), and ethionamide. Household members were screened for MDR-TB and none were found to have TB. Notification was made to the District Health Office and the National TB programme at the Ministry of Health, but both were unable to offer MDR-TB treatment; however, in collaboration with Médecins Sans Frontières, capreomycin, levofloxacin, cycloserine, prothionamide, and PAS, were made available, and the patient started treatment in March 2009. The patient received adherence counseling for MDR-TB, and his wife agreed to serve as a treatment supporter, which included picking up his medications from the clinic, ensuring directly observed therapy at home, and providing feedback about his condition to the clinic. During the initial treatment phase, a community volunteer nurse gave injections to the patient daily at his home. The patient was advised to stay at home.

Several infection control measures were put in place after confirmation of MDR-TB. The patient was given house quarantine, and his wife had a separate bedroom in the home until he received sputum conversion. The family was informed about TB transmission, and were encouraged to open windows and doors during the day. The patient was counseled on coughing etiquette, and visitors were restricted from accessing the patient's residence.

By July 2009, the sputum ZN smear was found to be negative, and the patient continued the initial treatments for another 4 months. By November 2009, the sputum ZN smear remained negative, and the patient continued on levofloxacin, cycloserine, prothionamide, and PAS for 12 more months. After achieving sputum conversion and starting on the continuation phase, the patient began attending the clinic again for his HIV care; antiretroviral therapy was continued for the duration of his MDR-TB treatment as per the Uganda National HIV/AIDS treatment guidelines and the World Health Organization [3,4]. Treatment for MDR-TB was completed in November 2010. The patient has been clinically stable since this time. He attained a peak CD4 cell count of 214 cells/mm [3] shortly before completing MDR-TB. He has continually had an undetectable HIV viral load. His T3, T4, and TSH are in normal ranges. Final sputum tests done in November 2011 at the National TB programme reference laboratory were negative, both for mycobacterium culture and sputum smear microscopy.

## Conclusions

This case report from Mildmay Uganda demonstrates successful treatment of MDR-TB using a home-based approach in Uganda. To our knowledge, this is the first reported home-based care of MDR-TB from East Africa. In many settings in Sub-Saharan Africa, MDR-TB is increasing in prevalence. However, as demonstrated with our case-report, the infrastructure to accurately diagnose MDR-TB is frequently lacking and the real extent of MDR-TB in East Africa at least, is unknown.

Our approach to applying home-based care strategies comes out of necessity rather than desirability. However, this may be an example where we have learned important lessons from our circumstances. Home-based care offers several important advantages over facility-based care, especially for HIV-positive patients who may be surrounded by other at-risk patients. First, by remaining at home they decrease their likelihood of infecting other HIV-positive patients. Second, by remaining at home they are surrounded by their families and social support networks. Evidence from South Africa indicates that mandating patients to remain away from their families can have devastating consequences and patients are unlikely to comply with this advice [5]. Third, by engaging family members as supportive individuals in their family's medical care, it is an opportunity to both educate the family and ensure retention and adherence to therapy. Evidence from an HIV program in Mozambique illustrates excellent outcomes using such an approach, with low mortality, low loss to follow-up and excellent adherence [6,7]. Finally, by being cared for at home, they reduce a burden on the hospital system and human resource challenges. This is an important burden in settings such as East Africa. We also recognize that there are limitations to a home-based care approach, such as the reliance on family and community, who may not always be available. We also recognize that since this is a case report, we are unable to directly compare the outcomes of the patient to another treated in-hospital.

The treatment of MDR-TB among HIV-positive patients in East Africa appears to be possible by employing a home-based care approach. Task-shifting hospital strategies into the community may be a long-term solution to current constraints in terms of hospital infrastructure and home resources.

## Consent

Written informed consent was obtained from the patient for publication of this case report.

## Competing interests

The authors declare that they have no competing interests.

## Authors' contributions

All authors contributed equally to the manuscript. All authors read andapproved the final manuscript.
